# ADMISSION AND CARE OF INDIVIDUALS WITH MENTAL ILLNESS IN MASSACHUSETTS NURSING HOMES: A PILOT STUDY

**DOI:** 10.1093/geroni/igz038.1237

**Published:** 2019-11-08

**Authors:** Adrita Barooah, Pamela Nadash

**Affiliations:** University of Massachusetts Boston, Boston, Massachusetts, United States

## Abstract

Due to the rising prevalence of mental illness in nursing homes (NHs), the US Congress passed the 1987 Pre-Admission Screening and Resident Review (PASRR) mandate, which aims both to limit inappropriate institutionalization of people with mental illness and to ensure that they are served appropriately when living in NHs. Although the PASRR is a federal mandate, states have considerable flexibility in implementing it, resulting in considerable variation across states. This study explores the Commonwealth of Massachusetts’ policies on admission and care of individuals with mental illness in NHs, focusing on implementation of PASRR regulations. Semi-structured phone interviews were conducted with key informants identified through purposive snowball sampling (N=8). Key informants included representatives from NHs, the State Mental Health Authority, state Medicaid office, and independent contractors and an academic expert. Data were analyzed using qualitative content analysis. Participants agreed that the PASRR tools efficiently identified and screened people with mental illness -- thus achieving PASRR’s first aim, but that the regulations did not successfully ensure appropriate services. Interviewees also identified a lack of services and options for diversion of people with mental illness into the community. Nursing home informants noticed a disconnect between the various supervising departments and felt instructions were unclear on the administration of the tool. This work builds a case for a national study to understand how PASRR implementation varies across states, resulting in variations in the proportion of people with mental illness admitted and served in NHs.

